# Low serum galectin-3 concentrations are associated with insulin resistance in patients with type 2 diabetes mellitus

**DOI:** 10.1186/1758-5996-6-106

**Published:** 2014-09-27

**Authors:** Tsuyoshi Ohkura, Youhei Fujioka, Risa Nakanishi, Hideki Shiochi, Keisuke Sumi, Naoya Yamamoto, Kazuhiko Matsuzawa, Shoichiro Izawa, Hiroko Ohkura, Etsuko Ueta, Masahiko Kato, Eiji Miyoshi, Shin-ichi Taniguchi, Kazuhiro Yamamoto

**Affiliations:** Division of Cardiovascular Medicine, Endocrinology and Metabolism, Department of Molecular Medicine and Therapeutics, Tottori University Faculty of Medicine, Yonago, Tottori Japan; Department of Regional Medicine, Tottori University Faculty of Medicine, Yonago, Tottori Japan; School of Health Science, Tottori University Faculty of Medicine, Yonago, Japan; Department of Molecular Biochemistry and Clinical Investigation, Osaka University Graduate School of Medicine, Suita, Japan

**Keywords:** Galectin-3, Insulin resistance, Type 2 diabetes mellitus

## Abstract

**Background:**

Galectin-3 is a family of soluble beta-galactoside-binding lectins that play many important regulatory roles in inflammation. Galectin-3-deficient mice have been shown to exhibit excess adiposity, hyperglycemia, insulin resistance and systemic inflammation. We investigated the association between serum galectin-3 and insulin resistance in patients with type 2 diabetes using a glucose clamp method.

**Methods:**

This was a cross-sectional study. Twenty patients (mean fasting plasma glucose 7.6 mmol/L, HbA1c 7.2%, BMI 28.1 kg/m^2^) underwent a meal tolerance test and glucose clamp test. Participants were given a test meal and plasma glucose and insulin were measured at 0, 30, 60, 120 and 180 min. The glucose disposal rate was measured during hyperinsulinemic-euglycemic glucose clamps. Serum galectin-3 levels were measured using the enzyme-linked immunosorbent assay method.

**Results:**

The mean serum galectin-3 level was 5103 pg/ml. Galectin-3 levels correlated significantly with the glucose disposal rate (R = 0.71, P < 0.001), fasting insulin (R = −0.56, P < 0.01), homeostasis model assessment for insulin resistance (R = −0.52, P < 0.05), and the insulin sensitivity index (R = 0.62, P < 0.005). Galectin-3 levels also positively correlated with the serum adiponectin level (R = 0.61, P < 0.05), but not with the high-sensitive C-reactive protein and interleukin-6 and −10.

**Conclusions:**

These results suggest that low levels of serum galectin-3 are associated with insulin resistance in patients with type 2 diabetes.

## Background

Galectin-3 is a family of soluble beta-galactoside-binding lectins that play important regulatory roles in inflammation [1]. Galectin-3 has been reported to be a predictor of prognosis of heart failure [2]. In type 2 diabetes, it was reported that systemic galectin-3 is elevated in obesity and is negatively correlated with glycated hemoglobin [3]. Galectin-3 was also increased in the serum of patients with elevated C-reactive protein. Compared with hepatic and systemic venous serum, galectin-3 was higher in the portal venous serum suggesting that the splanchnic region is a major site of galectin-3 synthesis. The lower levels of galectin-3 in the hepatic venous serum compared with the portal venous serum demonstrate that galectin-3 is removed by the hepatic system.

However, the pathophysiology of galectin-3 in diabetes is not well-known. One report, using an animal model, found that galectin-3+/+mice developed delayed and sustained hyperglycemia in streptozotocin-induced diabetes, mononuclear cellular infiltration and reduced insulin content of islets [4]. This was also accompanied by the expression of proinflammatory cytokines [4]. Galectin-3−/− mice were relatively resistant to diabetogenesis as evaluated by measurements of glycemia, quantitative histology and insulin. Thus, galectin-3 is involved in immune mediated beta cell damage and is required for diabetogenesis in this model of the disease. Another study has revealed that the role of endogenous galectin-3 in beta cell apoptosis in the inflammatory milieu that occurs during diabetes pathogenesis, implicates impairment of the mitochondrial apoptotic pathway as a key event in the protection from beta cell apoptosis in the absence of galectin-3 [5]. However, galectin-3 over-expression protected beta-cells from the cytotoxic effect of IL-1beta [6]. Moreover, Pang et al. showed that young (12-week-old) galectin-3 deficient mice fed a standard diet exhibited altered glucose homeostasis in the absence of obesity and associated abnormalities, thus suggesting a direct positive modulation of beta-cell function by galectin-3 independent of obesity-related inflammation [7]. A recent animal study reported that obese galectin-3−/− mice have increased body weight, total visceral adipose tissue, fasting blood glucose and insulin levels, homeostasis model assessment of insulin resistance (HOMA-IR), and markers of systemic inflammation compared with diet-matched wild-type animals [8]. Obesity induced by accelerated high fat diet in galectin-3-deficient mice was associated with systemic inflammation. Increased levels of interleukin (IL)-6 and reduced levels of IL-10 in the sera of obese galectin-3-deficient mice might contribute to amplified obesity-induced inflammation.

These results suggest that a low serum galectin-3 level is associated with hyperinsulinemia, insulin resistance and inflammation in type 2 diabetes. Furthermore, these reports suggest that a serum galectin-3 level is associated with beta cell function. Therefore, we hypothesize that low serum galectin-3 could be associated with insulin resistance and beta cell function in patients with type 2 diabetes. We measured serum galectin-3 levels in type 2 diabetes patients, and performed a glucose clamp method and a meal tolerance test (MTT) to evaluate insulin resistance and beta cell function.

## Methods

### Subjects

Twenty participants were subjected to a glucose clamp test and an MTT. Type 2 diabetes mellitus was diagnosed based on the criteria of the Japan Diabetes Society (JDS) [9]. The mean age of the patients was 53.6 years, mean BMI was 28.0 kg/m^2^, mean waist circumference was 95.8 cm, mean fasting plasma glucose (FPG) was 7.60 mmol/L and mean HbA1c was 7.25% (55 mmol/mol) (Table 1). Patients with ischemic heart disease, heart failure, pancreatic disease, liver disease, renal failure, or those taking diabetogenic medications such as corticosteroids were excluded from the study. Seven patients were on diet therapy alone and 13 were using oral hypoglycemic agents, including five on α-glucosidase inhibitors, five on dipeptidyl peptidase inhibitors, three on sulfonylurea, three on glinides and three on biguanides. None of the patients were using thiazolidinediones or insulin injections.

**Table 1:** **Patient characteristics**

***N***	20	Fasting insulin (pmol/L)	68.3 ± 41.6
Gender (male/female)	13/7	HOMA-IR	3.77 ± 2.33
Age (years)	53.6 ± 11.4	ISI	4.32 ± 2.26
BMI (kg/m^2^)	28.0 ± 3.8	Insulinogenic index	97.3 ± 117.1
Waist circumstance (cm)	95.8 ± 8.8	AUC glucose (mmol/L•h)	19.6 ± 3.5
FPG (mmol/L)	7.60 ± 1.18	AUC insulin (pmol/L•h)	448.0 ± 265.4
HbA1c (NGSP) (%)	7.25 ± 0.71	AUC insulin/AUC glucose	23.8 ± 14.3
(mmol/mol)	(55 ± 8)	hs-CRP (mg/dL)	0.16 ± 0.13
LDL-C (mmol/L)	3.33 ± 0.85	IL-6 (pg/ml)	2.96 ± 3.52
TG (mmol/L)	1.80 ± 0.89	IL-10 (pg/ml)	4.15 ± 2.93
HDL-C (mmol/L)	1.29 ± 0.37	adiponectin (μg/mL)	6.76 ± 3.57
GDR (mg · kg^−1^ · min^−1^)	5.33 ± 2.04	Proinsulin (pmol/L)	3.73 ± 3.32
Galectin-3 (pg/mL)	5103 ± 2239	Proinsulin/insulin ratio	0.09 ± 0.10

Data are means ± standard deviation.

*BMI*, body mass index; HbA1c, hemoglobin A1c; *NGSP*, National Glycohemoglobin Standardization Program; *FPG*, fasting plasma glucose; *LDL*-*C*, low-density lipoprotein cholesterol; *TG*, triglyceride; *HDL*-*C*, high-density lipoprotein cholesterol; *HOMA*-*IR*, homeostasis model assessment of insulin resistance; *ISI*, insulin sensitivity index; *AUC*, area under the concentration–time curve; *hs-CRP* high-sensitive C reactive protein; *IL*, interleukin; *GDR*, glucose disposal rate.

This study was approved by the Ethics Committee of the Faculty of Medicine, Tottori University and was conducted in compliance with the ethical principles of the Declaration of Helsinki. Informed consent was obtained from all of the patients using a procedure approved by the Ethics Committee.

### Meal tolerance test

After fasting for at least 12 h, the participants visited a morning clinic and consumed a test meal (total calories 460 kcal, carbohydrates 56.5 g, fat 18.0 g; protein 18.0 g) prepared by the JDS (JANEF E460F18, Kewpie Corporation, Tokyo, Japan) [10]. Plasma glucose and insulin were measured at 0 (fasting), 30, 60, 120, and 180 min after the meal. Plasma glucose was measured using the glucose oxidase method and plasma insulin using chemiluminescent immunoassays (CLIA) (human insulin CLIA kits, Kyowa Medix, Tokyo, Japan). Plasma insulin was defined as immunoreactive insulin (IRI). This method, using the meal tolerance test (MTT), is a well-established method in our hospital [11, 12].

HbA1c (JDS) was measured by high-performance liquid chromatography and was converted to National Glycohemoglobin Standardization Program (NGSP) values using the following certified equation, NGSP (%) = 1.02 × JDS (%) + 0.25% [13]. HbA1c (NGSP) values were also converted to International Federation of Clinical Chemistry (IFCC) values (mmol/mol) using the HbA1c converter developed by the National Institutes of Diabetes and Digestive and Kidney Diseases [14].

### Euglycemic–hyperinsulinemic clamp

Glucose clamp studies were performed 2 days after the MTT. The patients were examined in the morning after an overnight fast. An antecubital vein was cannulated to administer the infusate. A dorsal vein was cannulated and kept warm to facilitate venous sampling and provide arterialized venous blood. Using an artificial endocrine pancreas (STG 55; Nikkiso, Shizuoka, Japan), the euglycemic–hyperinsulinemic clamp was performed to determine insulin sensitivity in peripheral tissues [15]. A primed constant infusion of insulin (100 mU/m^2^ · min) and computer-controlled exogenous infusion of glucose solution were used to achieve steady-state plasma insulin levels and maintain plasma glucose levels at 5.2 mmol/L (95 mg/dL). Using the insulin infusion protocol as previously reported, the steady-state plasma insulin level was 1200 pmol/L in patients with type 2 diabetes [16, 17]. The steady-state glucose infusion rate (GIR) was calculated at 90–120 min, and the mean GIR during that time was used as a marker of peripheral insulin sensitivity. The mean GIR was defined as the glucose disposal rate (GDR). The glucose clamp method is a well-established procedure in our hospital [18]. Some of the data of the current study were already published in the previous study [18].

### Galectin-3, adiponectin, hs-CRP, IL-6, IL-10 and proinsulin assays

An enzyme-linked immunosorbent assay (ELISA) kit was used for measuring galectin-3 (Human Galectin-3 Assay Kit, Immuno-Biological Laboratories Co., Gunma, Japan) [19], plasma adiponectin (human adiponectin ELISA kit, Otsuka, Tokyo, Japan), plasma high-sensitive C reactive protein (hs-CRP) (human hs-CRP ELISA kit, Otsuka, Tokyo, Japan), plasma interleukin-6 (IL-6) and interleukin-10 (IL-10) (human IL-6, IL-10 Quantikine ELISA, R & D Systems, Inc., Minneapolis, USA) and plasma proinsulin (human intact proinsulin ELISA kit, Biovendor, Heidelberg, Germany).

### Calculation of insulin resistance and secretion indices

HOMA-IR was calculated by FPG (mmol/L) × fasting IRI (F-IRI pmol/L)/135 [20]. The insulin sensitivity index (ISI) was calculated by 10,000/√{[FPG (mmol/L) × F-IRI (pmol/L)] × [mean glucose × mean insulin during MTT]} [21]. Insulinogenic index was measured by [insulin (pmol/L) at 30 min – insulin (pmol/L) at 0 min]/[glucose (mmol/L) at 30 min – glucose (mmol/L) at 0 min] [22].

### Statistical analyses

Data are expressed as means ± standard error of the mean. The area under the curve (AUC) was calculated according to the trapezoidal rule. Correlations between parametric clinical variables and the galectin-3 were determined by Pearson’s correlation analysis. Multiple regression analysis was performed to examine an influencing factor of the serum galectin-3 level, the independent variables were: age, gender, BMI, waist circumference, FPG, HbA1c, fasting insulin, HOMA-IR, Insulinogenic Index, GDR, hs-CRP, IL-6, IL-10, proinsulin/insulin ratio, adiponectin, and triglyceride (TG). Values of P <0.05 were considered significant. SPSS software version 15.0 (SPSS, Chicago, IL, USA) was used for all analyses.

## Results

**Figure 1:**
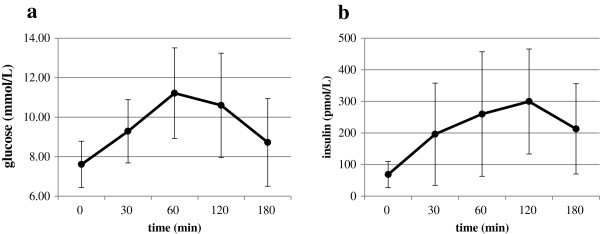
**Plasma glucose and insulin responses during the meal tolerance test.** Plasma glucose response during the meal tolerance test **(a)**, and insulin response **(b)** during the meal tolerance test.

Serum galectin-3 level of all participants was 5103 pg/mL (Table 1). The hs-CRP was 0.16 mg/d and adiponectin was 6.76 μg/mL. The FPG result of the MTT was 7.6 mmol/L, and the 2 h postprandial glucose was 10.6 mmol/L (Figure 1-a). Fasting insulin was 68.3 pmol/L and the 2 h postprandial insulin was 299.6 pmol/L (Figure 1-b). HOMA-IR was 3.77, ISI was 4.32 and the insulinogenic index was 97.3 (Table 1). During the steady state of glucose clamps, GDR was 5.33 mg · kg^−1^ · min^−1^ and the insulin level was 1159 ± 411 pmol/L. All results are given as means.

**Figure 2:**
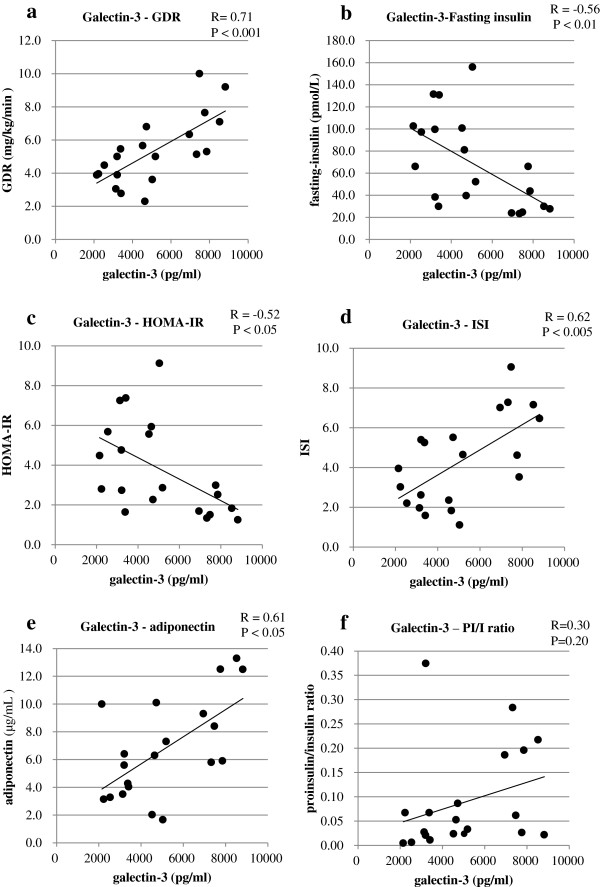
**Correlation between galectin**-**3 and other parameters.** Correlations between galectin-3 and GDR **(a)**, fasting insulin **(b)**, HOMA-IR **(c)**, ISI **(d)**, adiponectin **(e)**, and proinsulin/insulin (PI/I) ratio **(f)** were calculated by simple regression analysis.

Galectin-3 level strongly correlated with the GDR in the glucose clamp method (R = 0.71, P <0.001) (Table 2, Figure 2-a). Galectin-3 level negatively correlated with fasting insulin (R = −0.56, P <0.01) (Figure 2-b), and HOMA-IR (R = −0.52, P <0.05) (Figure 2-c). Galectin-3 level also positively correlated with the ISI in MTT (R = 0.62, P <0.005) (Figure 2-d) and with adiponectin (R = 0.61, P <0.05) (Figure 2-e). The AUC of glucose did not correlate with galectin-3 (R = −0.02, P = 0.92). The AUC of insulin and the ratio of AUC-insulin/AUC-glucose had a tendency to correlate with galectin-3, but this was not significant (R = −0.41, P = 0.06; R = −0.44, P = 0.05, respectively) (Table 2). The insulinogenic index had a tendency for negative correlation with galectin-3, but was not significant (R = −0.22, P = 0.33) (Table 2). The proinsulin/insulin ratio had a tendency for positive correlation with galectin-3, but again, was not significant (R = 0.30, P = 0.20) (Table 2, Figure 2-f). There were no significant correlations between galectin-3 and FPG, HbA1c, BMI, waist circumstance, lipid profiles, hs-CPR, IL-6 and IL-10 (Table 2).

**Table 2:** **Correlation coefficients between serum galectin**-**3 concentrations and other parameters**

Correlation of galectin-3 with	Pearson correlation	P value
GDR	0.71	< 0.001
Fasting insulin	−0.56	< 0.01
HOMA-IR	−0.52	< 0.05
ISI	0.62	< 0.005
Insulinogenic Index	−0.22	0.33
AUC glucose	−0.02	0.92
AUC insulin	−0.41	0.06
AUC insulin/AUC glucose	−0.44	0.05
hs-CRP	−0.25	0.27
IL-6	0.08	0.75
IL-10	−0.15	0.53
Proinsulin/insulin ratio	0.30	0.20
adiponectin	0.61	< 0.05
BMI	−0.17	0.48
waist circumference	−0.24	0.29
FPG	0.11	0.61
HbA1c	−0.10	0.66
LDL-C	0.27	0.23
TG	−0.14	0.52
HDL-C	0.28	0.21

Correlation coefficients were determined using Pearson’s product moment correlation coefficient test.

*GDR*, glucose disposal rate; *HOMA*-*IR*, homeostasis model assessment of insulin resistance; *ISI*, insulin sensitivity index; *AUC*, area under the concentration–time curve; *hs*-*CRP*, high-sensitive C reactive protein; *IL*, interleukin; *BMI*, body mass index; *FPG*, fasting plasma glucose; *LDL*-*C*, low-density lipoprotein cholesterol; *TG*, triglyceride; *HDL*-*C*, high-density lipoprotein cholesterol.

Using multiple regression analysis, we examined influence factors of serum galectin-3 level. The independent variables were: age, gender, BMI, waist circumference, FPG, HbA1c, fasting insulin, HOMA-IR, Insulinogenic Index, GDR, hs-CRP, IL-6, IL-10, proinsulin/insulin ratio, adiponectin, and TG. The significant standard partial regression coefficient was GDR 0.898 (P < 0.05) (Table 3).

**Table 3:** **Multiple regression analysis to examine an influencing factor of the galectin**-**3**

R^2^ = 0.767 R = 0.876	Partial regression coefficients	Standard partial regression coefficients	P value
Age	0.329	0.055	0.93
Gender	−0.007	0.503	0.38
BMI	−1.667	0.723	0.17
Waist circumference	−0.2481	−0.871	0.05
FPG	0.125	−0.829	0.08
HbA1c	−0.102	0.080	0.89
Fasting insulin	−0.564	−0.837	0.07
HOMA-IR	0.023	−0.098	0.337
Insulinogenic Index	0.402	−0.066	0.480
GDR	0.713	0.898	0.03
hs-CRP	−0.257	0.355	0.55
IL-6	−0.075	−0.196	0.75
IL-10	−0.146	0.274	0.65
Proinsulin/insulin ratio	0.297	0.833	0.08
adiponectin	0.614	−0.394	0.51
TG	−0.110	−0.266	0.66

The independent variables were: age, gender, BMI, waist circumference, FPG, HbA1c, fasting insulin, HOMA-IR, Insulinogenic Index, GDR, hs-CRP, IL-6, IL-10, proinsulin/insulin ratio, adiponectin, and TG. The significant standard partial regression coefficient was GDR 0.898 (P < 0.05).

BMI, body mass index; FPG, fasting plasma glucose; HOMA-IR, homeostasis model assessment of insulin resistance; GDR, glucose disposal rate; hs-CRP, high-sensitive C reactive protein; IL, interleukin; TG, triglyceride.

## Discussion

This study indicates that low serum galectin-3 concentrations strongly correlates with insulin resistance and hyperinsulinemia, evaluated by glucose clamp method, HOMA-IR, and ISI. Another study reported that obese galectin-3 knockout mice had increased fasting blood glucose and insulin levels, HOMA-IR and markers of systemic inflammation compared with diet-matched wild type animals [8]. Our study indicates that low levels of serum galectin-3 in patients with type 2 diabetes have insulin resistance and hyperinsulinemia similar to galectin-3 knockout mice. Although the area under the curve for glucose, for insulin and for the ratio insulin/glucose was not significantly associated with galectin-3 levels, there were significant associations between galection-3 and GDR, HOMA-IR, and ISI. These results suggested that the concentration of galectin-3 mainly affects insulin resistance rather than glucose levels.

Furthermore, galectin-3 was strongly correlated with adiponectin, which is an insulin sensitizer molecule. Pang et al. demonstrate the development of excess adiposity and systemic inflammation in galectin-3 deficiency that was associated with impaired fasting glucose levels and reduced adipose tissue expression of adiponectin and PPARγ [7]. These results suggest that the concentrations of galectin-3 and adiponectin are low in type 2 diabetes patients with insulin resistance, indicating that the combination of low galectin-3 and adiponectin induce strong insulin resistance.

An animal study reported that CRP and IL-6 were significantly increased in the sera of galectin-3 deficient mice given a high-fat diet, whereas the levels of IL-10 were significantly decreased compared with the diet-matched wild type mice [8]. In our study, hs-CRP, IL-6 and IL-10 were not correlated with galectin-3. IL-10 has a protective role in type 2 diabetes by increasing insulin sensitivity in skeletal muscle [23]. We presumed that low levels of plasma galectin-3 and IL-10 reflect insulin sensitivity in skeletal muscle, because the glucose clamp technique mainly reflects insulin sensitivity in skeletal muscle. However, there was no correlation between IL-10 and galectin-3 levels in this study. Further investigations are needed because the pathophysiology of type 2 diabetes patients may be different from that of galectin-3 knockout mice. We consider that galectin-3 is associated with adiponectin rather than inflammation in patients with type 2 diabetes.

In our study, galectin-3 had a weak tendency towards negative correlation with the insulinogenic index, and a positive correlation with the proinsulin/insulin ratio. The proinsulin/insulin ratio is a marker of beta cell stress [12]. Galectin-3−/− mice were relatively resistant to diabetogenesis as evaluated by glycemia, quantitative histology and insulin content in streptozotocin induced diabetes [4]. These results imply that low levels of serum galectin-3 induce insulin resistance and hyperinsulinemia, but also may protect beta cell function. However, galectin-3 over-expression protected beta-cells from the cytotoxic effect of IL-1beta [6]. Moreover, young (12-week-old) galectin-3 deficient mice fed a standard diet exhibited altered glucose homeostasis in the absence of obesity and associated abnormalities [7]. Thus suggesting a direct positive modulation of beta-cell function by galectin-3 independent of obesity-related inflammation. In this study, there was no significant association, because one patient with low galectin-3 showed high PI/I ratio. The small number of our study has limitations, further investigations are needed. As the multiple regression analysis shows, we consider that galectin-3 is associated with insulin resistance rather than insulin secretion in patients with type 2 diabetes.

Galectin-3 is also one of the pattern recognition receptors that bind and mediate the degradation of modified lipoproteins and advanced glycation end products (AGE) [24]. In contrast to other receptors for AGE, galectin-3 protects from AGE-induced tissue injury. Therefore, galectin-3 ablation accelerates AGE-induced atherogenesis [25]. These results suggest that low levels of serum galectin-3 also induce atherosclerosis, and therefore, galectin-3 is an important molecule in type 2 diabetes.

Our study has several limitations, including the small number of patients and the variable nature of the medications for diabetes used by the study participants. Thus, our results require confirmation by a larger study. It is possible that the different medications used by the subjects modified the insulin responses in the MTT. For instance, metformin has been associated with lower systemic galectin-3 [3]. In our study, three patients were treated with metformin, which could have affected galectin-3 levels. A control group of insulin resistant non-diabetic subjects would be helpful in this regard, we are investigating a control group of insulin resistant non-diabetic subjects now. Despite these limitations, we consider that our study contributes to better understanding of the pathophysiology of type 2 diabetes.

## Conclusion

Our results suggest that low levels of serum galectin-3 are associated with insulin resistance in patients with type 2 diabetes.

## Abbreviations

AUC: Area under the curve
AGE: Advanced glycation end product
ELISA: Enzyme-linked immunosorbent assay
FPG: Fasting plasma glucose
GDR: Glucose disposal rate
GIR: Glucose infusion rate
HOMA-IR: Homeostasis model assessment for insulin resistance
hs-CRP: high-sensitive C-reactive protein
IL: Interleukin
IRI: Immunoreactive insulin
ISI: Insulin sensitivity index
JDS: Japan Diabetes Society
MTT: Meal tolerance test.
